# Measurement of blood acetoacetate and β-hydroxybutyrate in an automatic analyser

**DOI:** 10.1155/S1463924601000086

**Published:** 2001

**Authors:** Amparo Galán, Josém. Hernández, Orlando Jimenez

**Affiliations:** Clinical Biochemistry Service Hospital Universitario Germans Trias i Pujol Carretera del Canyet s/n Badalona Barcelona Spain

## Abstract

g-hydroxybutyrate and acetoacetate as well as lactate and pyruvate are intermediary metabolites normally present in blood. The g-hydroxybutyrate/acetoacetate ratio is an expression of the mitochondrial oxido-reduction state. This ketone body ratio can provide a clue to diagnosis and metabolic status in congenital errors of the electron transport chain and pyruvate metabolism. The standardization of these analytical procedures improves the interpretation of the results helping in the difficult diagnosis of mitochondrial diseases in children. This study describes an adaptation to a Dimension R 2 L (Dade Behring, Newark, Delaware, USA) automatic analyser for a method to measure blood ketone bodies (g-hydroxybutyrate and acetoacetate). The method allows the metabolites to be measured directly in nondeproteinized plasma (fluoride/ethylenediaminetetraacetic acid). This adaptation simplifies the analytical procedure and limits the turnaround time to 20 minutes. With a sample volume of 200 μ l metabolite concentrations ranging from 12 to 1300 μ molL^−1^ of g-hydroxybutyrate and from 10 to 450 μ molL^−1^ of acetoacetate may be measured with a reliable analytical response.


Journal of Automated Methods & Management in Chemistry, Vol. 23, No. 3 (May–June 2001) pp. 69–76

### Measurement of blood acetoacetate and β-hydroxybutyrate in an automatic analyser

**Amparo Galán[1], José M. Hernández and Orlando Jimenez**

*Clinical Biochemistry Service, Hospital Universitario Germans Trias i Pujol, Carretera del Canyet s/n, Badalona, Barcelona, Spain. [1] e-mail: agalan@ns.hugtip.scs.es*

*β-hydroxybutyrate and acetoacetate as well as lactate and pyruvate are intermediary metabolites normally present in blood. The β-hydroxybutyrate/acetoacetate ratio is an expression of the mitochondrial oxido-reduction state. This ketone body ratio can provide a clue to diagnosis and metabolic status in congenital errors of the electron transport chain and pyruvate metabolism. The standardization of these analytical procedures improves the interpretation of the results helping in the difficult diagnosis of mitochondrial diseases in children. This study describes an adaptation to a Dimension $R \times L$ (Dade Behring, Newark, Delaware, USA) automatic analyser for a method to measure blood ketone bodies (β-hydroxybutyrate and acetoacetate). The method allows the metabolites to be measured directly in nondeproteinized plasma (fluoride/ethylenediaminetetraacetic acid). This adaptation simplifies the analytical procedure and limits the turnaround time to 20 minutes. With a sample volume of 200 μl metabolite concentrations ranging from 12 to 1300 $\mu mol\,L^{-1}$ of β-hydroxybutyrate and from 10 to 450 $\mu mol\,L^{-1}$ of acetoacetate may be measured with a reliable analytical response.*

### Introduction

Ketone bodies are produced in the liver, mainly from the oxidation of fatty acids, and are exported to peripheral tissues for use as an energy source. The term 'ketone bodies' refers to three molecules, acetoacetate, β-hydroxybutyrate and acetone. Acetoacetate and β-hydroxybutyrate transport energy from the liver to other tissues and acetone is generated by spontaneous decarboxylation of acetoacetate [1]. The presence of ketosis may be normal or pathologic. Normally ketosis implies that the lipid energy metabolism has been activated and the pathway of lipid degradation is intact. Ketosis is a physiological finding in many circumstances such as during fasting, after prolonged exercise or after a high fat diet. However, as a general rule, hyperketosis, at a level that produces metabolic acidosis is not physiologic. Pathological causes of ketosis include diabetes, childhood hypoglycaemia, corticosteroid or growth hormone deficiency, intoxication with alcohol or salicylates and several inborn errors of metabolism, as well as multiple organ failure.

Ketosis with an abnormal elevation of the β-hydroxybutyrate/acetoacetate ratio implies a disorder of the intramitochondrial redox state [2]. These situations may be present in inborn metabolic diseases that affect fuel homeostasis such as disorders of the Krebs cycle, disorders of the mitochondrial respiratory chain, ketogenesis or ketolysis [3–5]. In these cases the evaluation of the lactate/pyruvate ratio together with the β-hydroxybutyrate/acetoacetate ratio are the tools of first choice in determining the cytoplasmatic and mitochondrial oxido-reduction state respectively of the individual [4]. The first application of these assays is in the screening for the diagnosis of mitochondrial cytopathies [6]. The determination of these ratios in the basal state or following an effort test [4, 7], may diagnose or discount a mitochondrial cytopathy. If these test results are normal, a biopsy may be avoided.

Spectrophotometric determinations of circulating acetoacetate and hydroxybutyrate have been reported [8, 9] and adapted to several automatic analysers [10–14]. The quantification of ketone bodies presents technical (manual preparation of reagents, complexity of the pre-analytical phase, long analytical response times) and diagnostic (intraindividual variations, wide reference ranges, need for support from other biochemical parameters for result interpretation) interpretation difficulties [15].

With the aim of standardizing the analytical procedures and facilitating their application as routine techniques we have adapted the methods proposed by Vassault *et al.* [16] to measure ketone bodies on a Dimension $R \times L$ (Dade Behring, Newark, Delaware, USA) automatic analyser. The study was performed on two types of sample: deproteinized plasma with perchloric acid and afterwards alkalinized, and plasma obtained directly with fluoride/ethylenediaminetetraacetic acid (EDTA).

### Material and methods

### *Principle of the method*

The two assays are based on end-point reactions catalysed by β-hydroxybutyrate dehydrogenase at 37 °C and different pH conditions. The generation or consumption of reduced nicotinamide ademine dinucleotide (NADH) is measured by changes in absorbance at 340 nm:

$$\beta\text{-hydroxybutyrate} + \text{NAD}^- \xleftrightarrow[\text{dehydrogenase}]{\beta-\text{hydroxybutyrate}} \text{acetoacetate} + \text{NADH}^- + \text{H}^+$$

To complete the β-hydroxybutyrate reaction a pH of 9.5 is necessary while the acetoacetate assay requires a pH of 7.

http://www.tandf.co.uk/journals
DOI: 10.1080/14639240010026084

*Table 1. Settings for β-hydroxybutyrate determination by the dimension $R \times L$ analyser.*

Reagent and sample delivery

| Setting | Sample (S1) | First reagent (R1) (NAD 168 mM) | Second reagent (R2) (Tris 0.1M pH 9) | Third reagent (R3) (HBDH 15 U ml$^{-1}$) |
|---|---|---|---|---|
| Delivery time (s) | 0.0 | −60.0 | 65.3 | 125.3 |
| Volume delivery (μl) | 60 | 20.0 | 245.0 | 12.0 |
| Chase delivery (μl) | 0.0 | 0.0 | 20.0 | 20.0 |
| Mix | None | None | Moderate | Moderate |

Photometric readings and calculation

| | |
|---|---|
| Mode | Absorbance |
| Standard curve | Logit |
| Measuring filter | |
| First filter | 340 nm |
| Secondary filter | 700 nm |
| Time of photometric readings | |
| P1 | 110 s |
| P2 | 410 s |
| Calculation mode | Endpoint |
| Dilution | $C = A \times (-0.915) + B \times (1.0)$ |

*Reagents and work solutions*

β-Hydroxybutyrate

- Reagent 1: 168 mmol L$^{-1}$: nicotinamide adenine dinucleotide (NAD) (Roche catalogue 127841) in distilled water. Stable for 6 months when frozen at −20 °C in aliquots.
- Reagent 2: 0.1 mol L$^{-1}$ tris buffer (tris(hydroxymethyl)-aminomethane, Merck catatalogue 8382) adjusted to pH 9.5 with HCl.
- Reagent 3: β-hydroxybutyrate dehydrogenase (EC1.1.1.30) (HBDH Roche catalogue 127841; specific activity: 3 U mg$^{-1}$ (25 °C). Stable at 4 °C.
- β-hydroxybutyrate standards to calibrate the method: D,L β-hydroxybutyric acid, sodium salt (Sigma H6501). Prepare 0, 50, 250, 500 and 1250 μmol L$^{-1}$ solutions in distilled water. Stable for 6 months when frozen at −20 °C in aliquots.

Acetoacetate

- Reagent 1: 4 mmol L$^{-1}$ NADH (Roche catalogue 128023) in distilled water. Stable for 6 months when frozen at −20 °C in aliquots.
- Reagent 2: 0.1 mol L$^{-1}$ tris buffer (tris(hydroxymethyl)-aminomethane, Merck catalogue 8382) adjusted to pH 7.
- Reagent 3: β-hydroxybutyrate dehydrogenase (EC1.1.1.30) (Roche catalogue 127841); specific activity 3 U mg$^{-1}$ (25 °C). Stable at 4 °C.
- Acetoacetate standards to calibrate the method: acetoacetic acid salt (Sigma, catalogue A-8509). Prepare 0, 50, 100, 200 and 400 μmol L$^{-1}$ solutions in distilled water. Stable for 4 weeks when frozen at −20 °C in aliquots.

*Reagents for specimen preparation*

For deproteinized plasma samples: 1 mol L$^{-1}$ perchloric acid (Merck catalogue 519) and 0.7 mol L$^{-1}$ tripotassium phosphate (Merck catalogue 5102).

For plasma samples: fluoride/EDTA (Roche catalogue 243710).

*Automated assay*

The settings used on the Dimension $R \times L$ analyser are listed in tables 1 and 2.

*Calibration method*

Calibration was with β-hydroxybutyrate and acetoacetate standards described in *Reagents and work solutions* above. β-Hydroxybutyrate was calibrated following a logarithmic reaction and acetoacetate a linear reaction.

*Subjects*

Reference values were calculated from 60 samples obtained from apparently normal children, and were sent to our laboratory for analytical control before minor surgical interventions were carried out on the patients. Blood samples were obtained after 12 hours of fasting. The age range of the children was 1 month to 14 years. The specimens were assayed the same day as the blood samples were obtained.

20 of these values were used to study the comparison between plasma and deproteinized plasma samples. 20 apparently normal subjects (age range 20–60 years), 20 patients with diabetic ketosis and another 20 patients with metabolic disorders were also included in the study.

*Specimens*

The blood specimens (1 ml) were added immediately to tubes containing 1 ml cold 1 mol L$^{-1}$ perchloric acid. The samples were mixed and centrifuged (3000 × g for 10 minutes). The deproteinized supernatants were stored at −20 °C until analysis. The supernatants were adjusted to

*Table 2. Settings for acetoacetate determination by the Dimension $R \times L$ analyser.*

Reagent and sample delivery

| Setting | Sample (S1) | First reagent (R1) (NAH 4 mM) | Second reagent (R2) (Tris 0.1 M pH 7) | Third reagent (R3) (HBDH 15 U ml$^{-1}$) |
|---|---|---|---|---|
| Delivery time (s) | 0.0 | −60.0 | 65.3 | 125.3 |
| Volume delivery (µl) | 95 | 16.0 | 95.0 | 5.0 |
| Chase delivery (µl) | 0.0 | 0.0 | 20.0 | 20.0 |
| Mix | None | None | Moderate | Moderate |

Photometric readings and calculation

| | |
|---|---|
| Mode | Absorbance |
| Standard curve | linear |
| Measuring filter | |
| First filter | 340 nm |
| Secondary filter | 700 nm |
| Time of photometric readings | |
| P1 | 110 s |
| P2 | 560 s |
| Calculation mode | Endpoint |
| Dilution | $C = A \times (-0.900) + B \times (1.0)$ |

pH 7 with 0.7 mol L$^{-1}$ tripotassium phosphate, stored for 15 minutes at 4 °C and then centrifuged. The alkalinized supernatants were used to measure the ketone bodies.

To obtain plasma samples 1 ml blood was added to tubes containing 1 drop of fluoride/EDTA and then mixed. The supernatants obtained after centrifuging at 3000 × g for 10 minutes were stored at −20 °C and used to measure the ketone bodies.

### *Quality control*

β-Hydroxbutyrate and acetoacetate standards, at two levels, were always assayed with patient samples first at one level and at the end of the batch at the other level. The assay was considered to be correct if the standard values were within ±10% of the assigned value.

### *Evaluation of the method*

The analytical interval and the detection limit of the methods were evaluated following the directions of the Socité Franµaise de Biologie Clinique [17]. To calculate the analytical interval the following standard aqueous solutions were used: 2500, 2000, 1250, 500, 250, 100, 25, 0.1 µmol L$^{-1}$ of D,L-β-hydroxybutyric acid, sodium salt, and 600, 500, 250, 100, 50, 25, 10, 5, 0.1 µmol L$^{-1}$ acetoacetic acid salt. The detection limit ($L_d$) was established following the processing of 10 specimens of distilled water. With the mean ($m_d$) and the standard deviation (SD) the detection limit for an $\alpha$ and $\beta$ risk of 5% ($K = 5.185$) was calculated ($L_d = m_d + K$ SD) [17].

The recommendations of the European Committee for Clinical Laboratory Standards (ECCLS) were followed for the study of the imprecision and inaccuracy of the method [18]. 50,100 and 200 µmol L$^{-1}$ hydroxybutyrate and acetoacetate standard aqueous solutions were used.

To determine the analytical recovery of the methods, increasing quantities of β-hydroxybutyrate or acetoacetate were added to different aliquots of the pool of sera. 430, 700 and 1000 µmol L$^{-1}$ hydroxybutyrate and 100, 200 and 400 mol L$^{-1}$ acetoacetate were the standard aqueous solutions used. The dilution effect was corrected by adding the same volume of saline solution to unaltered samples.

The reference values of the methods were obtained following the recommendations of the Panel of Experts of the International Federation of Clinical Chemistry on reference values [19].

### *Sample evaluation : deproteinized plasma or not*

β-Hydroxybutyrate and acetoacetate were assayed in 80 patients in replicate using as samples plasma obtained directly with fluoride/EDTA and deproteinized plasma with perchloric acid. Comparison studies were performed among the results from the two different samples.

### *Statistical methods*

The mean, standard deviation and coefficient of variation for studying the accuracy, imprecision and detection limit were obtained. The Student's $t$ test was applied to compare the types of sample. Regression analyses were applied to obtain the analytical interval. The Mann–Whitney test was applied to establish the statistical differences between groups. To calculate the reference values of the method, the 2.5, 50 and 97.5 percentiles were used.

### Results

### *Detection limit and analytical interval of methods*

The detection limit established was 12 µmol L$^{-1}$ for β-hydroxybutyrate and 6 µmol L$^{-1}$ for acetoacetate. The analytical interval of the methods ranged from 12 to 1300 µmol L$^{-1}$ for β-hydroxybutyrate and from 10 to 450 µmol L$^{-1}$ for acetoacetate. Figure 1 shows the slope

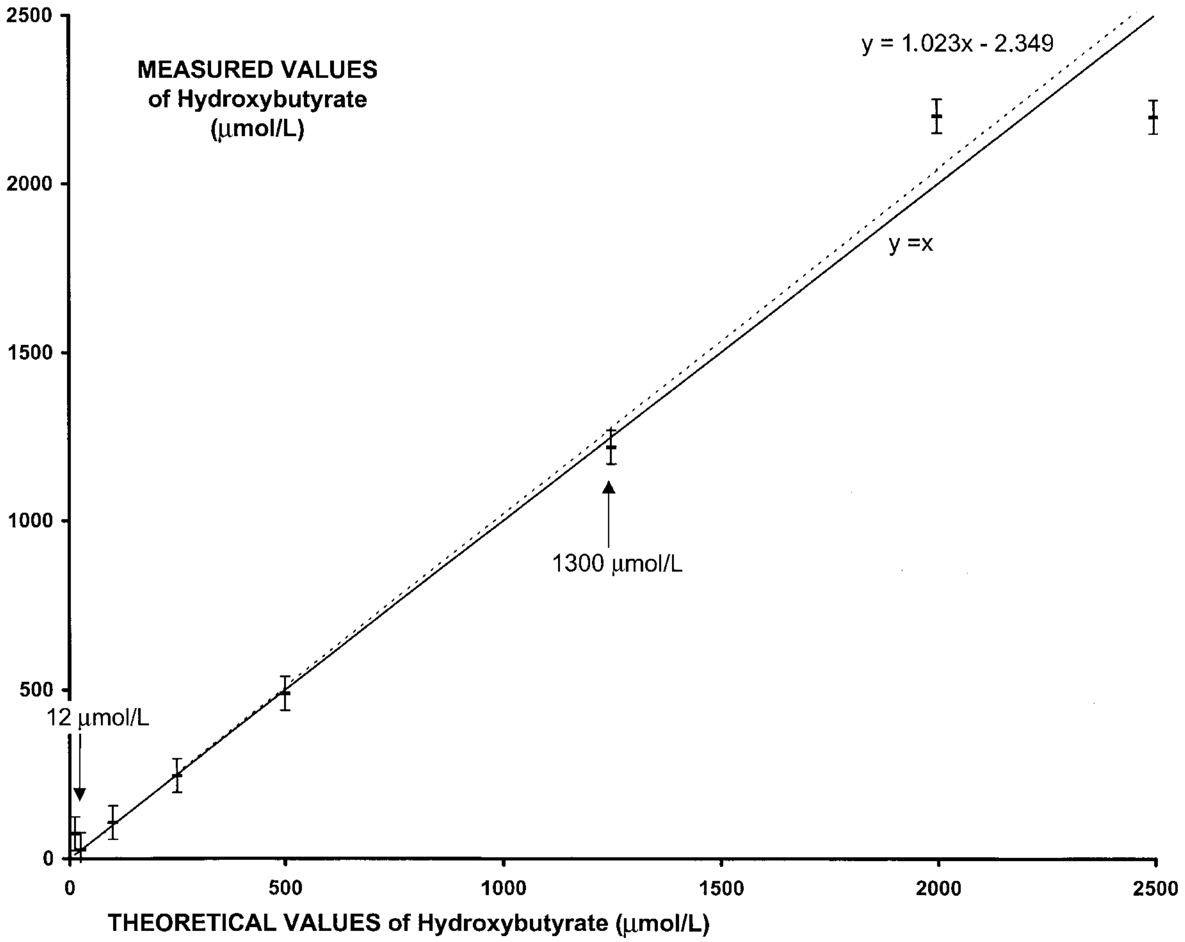

*Figure 1. Analytical interval for $\beta$-hydroxybutyrate. (—), Reference line $y = bx$ $(b = 1)$; (-----) regression line $(y = 1.023x - 2.349)$; (Ī) mean and two standard deviations of each point. The mean values of the points found and their two standard deviations coincide with the reference line from 12 to 1300 $\mu mol\ L^{-1}$.*

of linear regression of the least squares ($y = 1.023 \times -2.349$; $r = 0.989$) of the reference hydroxybutyrate standards processed in triplicate over 3 consecutive days, and the values found. The linear regression for the acetoacetate standard was $y = 1.020 \times -3.01$ ; $r = 0.976$) (figure 2).

*Imprecision and inaccuracy.* Table 3 demonstrates the within-run and between-run coefficients of variation of the aqueous standards of β-hydroxybutyrate and acetoacetate at three concentration levels, as well as those of the serum specimen. The percentages of inaccuracy with respect to the theoretical value (table 4) did not exceed 10%.

*Analytical recovery study.* Analytical recovery ranged from 97 to 104% for hydroxybutyrate and 98 to 103% for acetoacetate (table 5).

*Reference values.* Table 6 contains the reference values for the studied population which was divided into two groups (1 month to 7 years and 7 to 14 years). Statistical differences between the two groups were found for the β-hydroxybutyrate ($p = 0.032$) and acetoacetate ($p = 0.041$) results, but no differences were found for the β-hydroxybutyrate/acetoacetate ratio ($p = 0.200$).

*Comparison between plasma and deproteinized plasma samples.* No statistical differences were found for the concentrations of β-hydroxybutyrate ($t = -0.287$, $p = 0.775$) and acetoacetate ($t = -0.430$, $p = 0.669$) using deproteinized plasma with perchloric and non-deproteinized plasma (fluoride/EDTA) (table 7).

### Discussion

The determination of levels of blood ketone bodies is an infrequent type of analysis in clinical biochemistry laboratories. There are several reasons for this. Firstly, clinical interpretation of these results is difficult because of intraindividual variations, thereby making it difficult to establish reference values. Secondly, there are many physiological situations in which ketosis may be produced, thus to achieve a correct diagnosis of hyperketosis, other metabolites, such as glucose, lactate, pyruvate or amino acids, should be measured simultaneously. Therefore, use of blood ketone bodies is restricted to some concrete and difficult diagnoses. An example is the investigation of inborn errors of intermediary metabolism, especially affecting energetic homeostasis (defects of the mitochondrial respiratory chain, β-oxidation, ketogenesis and ketolysis). Since the incidence of these diseases is low, few manufacturers have been motivated to elaborate commercial kits for measuring these metabolites and even less to implement them in an automatic

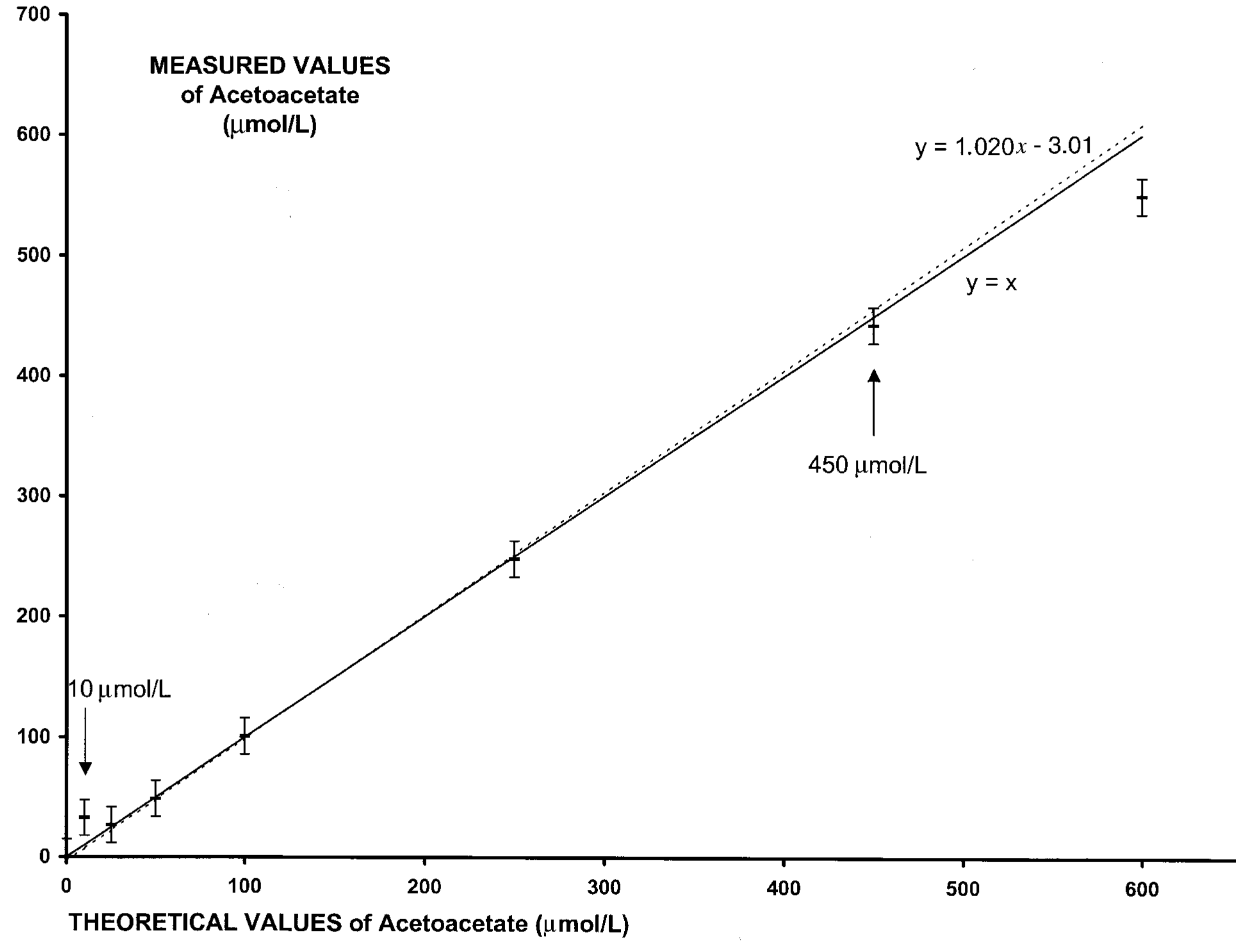

*Figure 2. Analytical interval for acetoacetate. (—), Reference line $y = bx$ ($b = 1$); (-----) regression line ($y = 1.020x - 3.01$); (Ī) mean and two standard deviations of each point. The mean values of the points found and their two standard deviations coincide with the reference line from 10 to 450 μmol $L^{-1}$.*

*Table 3. Imprecision of the methods.*

β-Hydroxybutyrate

| Concentration level | N | Within-run CV(%) | Between-run (CV%) |
|---|---|---|---|
| Aqueous β-hydroxybutyrate solution (50 μmol $L^{-1}$) | 20 | 6 | 9.5 |
| Aqueous β-hydroxybutyrate solution (100 μmol $L^{-1}$) | 20 | 5 | 9.1 |
| Aqueous β-hydroxybutyrate solution (200 μmol $L^{-1}$) | 20 | 4 | 7.3 |
| Serum pool (170 μmol $L^{-1}$) | 20 | 5.1 | — |
| Deproteinized plasma pool (110 μmol $L^{-1}$) | 20 | 5.2 | — |

Acetoacetate

| Concentration level | N | Within-run CV(%) | Between-run (CV%) |
|---|---|---|---|
| Aqueous acetoacetate solution (50 μmol $L^{-1}$) | 20 | 5 | 9.5 |
| Aqueous acetoacetate solution (100 μmol $L^{-1}$) | 20 | 4 | 9.1 |
| Aqueous acetoacetate solution (200 μmol $L^{-1}$) | 20 | 3 | 7.3 |
| Serum pool (96 μmol $L^{-1}$) | 20 | 4.5 | — |
| Deproteinized plasma pool (141 μmol $L^{-1}$) | 20 | 4.9 | — |

analyser. The manual practice of these assays does not favour their use since the preparation of the reagents and the assay itself are laborious, time-consuming, require high reagent and sample volumes, and give elevated imprecisions [8, 9].

Similar to several previous works [10–14], we have automated a spectrometric method [16] to measure ketone bodies in a Dimension $R \times L$ analyser, which is available 24 hours a day in the laboratory. With this adaptation we have improved notably the analytical reliability of our results in addition to saving time, and reagent and sample volume. This is an important point to take into account since most samples are of paediatric origin. Moreover, complementary analyses must be performed to achieve the diagnosis

*Table 4. Inaccuracy of methods.*

β-Hydroxybutyrate

| Theoretica concentrationl (μmol L$^{-1}$) | *N* | Within-run | | Between-run | |
|---|---|---|---|---|---|
| | | Mean found concentration (μmol L$^{-1}$) | Inaccuracy (%) | Mean found concentration (μmol L$^{-1}$) | Inaccuracy (%) |
| 50 | 20 | 53 | 6 | 47 | −6 |
| 100 | 20 | 101 | 1 | 105 | 5 |
| 200 | 20 | 198 | −1 | 186 | −7 |

Acetoacetate

| Theoretical concentration (μmol L$^{-1}$) | *N* | Within-run | | Between-run | |
|---|---|---|---|---|---|
| | | Mean found concentration (μmol L$^{-1}$) | Inaccuracy (%) | Mean found concentration (μmol L$^{-1}$) | Inaccuracy (%) |
| 50 | 20 | 48 | −4 | 53 | 6 |
| 100 | 20 | 98 | −2 | 92 | −8.0 |
| 200 | 20 | 195 | −2.5 | 215 | 7.5 |

*Table 5. Recovery study.*

β-Hydroxybutyrate

| Serum pool (μmol L$^{-1}$) | β-Hydroxybutyrate added (μmol L$^{-1}$) | Theoretical β-Hydroxybutyrate concentration (μmol L$^{-1}$) | Found β-Hydroxybutyrate concentration (μmol L$^{-1}$) | Recovery (%) |
|---|---|---|---|---|
| 200 | 430 | 630 | 655 | 104 |
| 200 | 700 | 900 | 873 | 97 |
| 200 | 1000 | 1200 | 1284 | 104 |

Acetoacetate

| Serum pool (μmol L$^{-1}$) | Acetoacetate added (μmol L$^{-1}$) | Theoretical acetoacetate concentration (μmol L$^{-1}$) | Found acetoacetate concentration (μmol L$^{-1}$) | Recovery (%) |
|---|---|---|---|---|
| 80 | 100 | 180 | 176 | 98 |
| 80 | 200 | 280 | 288 | 103 |
| 80 | 400 | 480 | 475 | 99 |

*Table 6. Reference values.*

| | 0–7 years ($n = 30$) | 7–14 years ($n = 30$) |
|---|---|---|
| β-Hydroybutyrate (μmol L$^{-1}$) | | |
| Range ($p_{2.5}, p_{97.5}$) | 20–700 | 20–500 |
| Mean value ($p_{50}$) | 60 | 40 |
| Acetoacetate (μmol L$^{-1}$) | | |
| Range ($p_{2.5}, p_{97.5}$) | 10–200 | 10–140 |
| Mean value ($p_{50)}$ | 45 | 31 |
| $\beta$-Hydroybutyrate/acetoacetate | | |
| Range ($p_{2.5}, p_{97.5}$) | 0.04–4.0 | 0.04–3.5 |
| Mean value ($p_{50}$) | 1.3 | 1.1 |

*Table 7. Comparison between deproteinized and non-deproteinized plasma as samples*

$\beta$-Hydroxybutyrate

| Sample | $n$ | Mean value ($\mu mol\ L^{-1}$) | Standard deviation ($\mu mol\ L^{-1}$) | Range ($\mu mol\ L^{-1}$) | Student's $t$ | $p$ | Pearson coefficient $r$ | Regression line |
|---|---|---|---|---|---|---|---|---|
| Deproteinized plasma ($x$) | 80 | 402.46 | 394.33 | 10–1290 | $-0.287$ | 0.775 | 0.991 | $y = 1.035x - 0.99$ |
| Plasma (fluoride/EDTA) non-deproteinized ($y$) | 80 | 400.46 | 377.58 | 10–1220 | | | | |

Acetoacetate

| Sample | $n$ | Mean value ($\mu mol\ L^{-1}$) | Standard deviation ($\mu mol\ L^{-1}$) | Range ($\mu mol\ L^{-1}$) | Student's $t$ | $p$ | Pearson coefficient $r$ | Regression line |
|---|---|---|---|---|---|---|---|---|
| Deproteinized plasma ($x$) | 80 | 154.45 | 134.97 | 10–450 | $-0.430$ | 0.669 | 0.988 | $y = 1.025x - 1.637$ |
| Plasma (fluoride/EDTA) non-deproteinized ($y$) | 80 | 153.26 | 130.02 | 10–430 | | | | |

and they can be processed simultaneously in the same analyser.

Another difficulty, from the technical point of view, in analysing ketone bodies derives from sample collection. Acetoacetate is an unstable metabolite [16] thus it is recommended that the blood be collected immediately after its withdrawal in an acid medium to be deproteinized as well as the samples be maintained in ice until processing. Other authors [13, 16], who usually determine lactate, pyruvate and ketone bodies in the same sample, recommend the use of deproteinized plasma with perchloric acid. We also perform our assays in this type of sample. However, we have observed that the results using plasma collected directly in fluoride/EDTA are similar to those obtained from deproteinized plasma with perchloric acid. On the other hand, statistically significant differences were found ($n = 80$, $t = 8.08$, $p < 0.001$) between using deproteinized plasma ($y$) and non deproteinized plasma (fluoride/EDTA) ($x$) in the quantification of pyruvate ($y = 1.74x + 4.62$; $r = 0.599$) (data not included in the study). We did not observe differences in the lactate values using these two types of sample ($n = 80$, $t = 0.118$, $p = 0.779$). Thus, if the four metabolites are to be measured in the same sample it is necessary to use deproteinized plasma in an acid medium after extraction because the pyruvate values may be incorrect. However, the ketone bodies may be evaluated directly in a serum sample which has been withdrawn with fluoride/EDTA with the aim of measuring its lactate for example. There are several advantages to working directly with non-deproteinized plasma. The possible dilution error (which falsifies the absolute value of the metabolites although not their ratios) is minimized. Additionally, the analytical procedure is reduced by 30 minutes. The present adaptation, especially using non-deproteinized plasma, simplifies the anaytical procedure and, although the preparation of reagents is somewhat laborious, the fact that they may be stored frozen, allows the assay to be performed with relative ease in emergency situations.

We have established our reference values for a population under the age of 14 years. Significant differences were found in two age groups for the two metabolites, but not for their ratios. These results have been reported by Artuch *et al.* [13]. Similar to other reports [13, 20], our reference values present a wide range of values. Many intraindividual variations may be due to the nutritional state of the patient.

In summary, this adaptation facilitates the measurement of sanguineous ketone bodies and provides a rapid and reliable method with low sample consumption and which may to be applied as a routine technique in an emergency laboratory. The main application of this method is to contribute to the diagnosis of mitochondrial diseases both in the first orientation as well as in subsequent provocative tests [6, 7].

### References

1. Guthrie, J. P. and Jordan, F., 1972, Amine-catalyzed decarboxylation of acetoacetic acid. The rate constant for decarboxylation of beta-immino acid. *J. Am. Chem. Soc.*, **94**, 9136.
2. Robinson, B. H., 1989, Lactic acidemia. In: C. R. Scriver, A. L. Beaudet, Ws. Sly and D. Valle (eds) *The Metabolic Basis of Inherited Disease* (New York: McGraw-Hill), p. 869.
3. Roth, K. S., 1991, Inborn errors of metabolism: the essentials of clinical diagnosis. *Clin. Pediatr.*, **30**, 183.
4. Munnich, A., Rötig, A., Chretien, D., Saudubray, J. M., Cormier, V. and Rustin, P., 1996, Clinical presentation and laboratory investigations in respiratory chain deficiency. *Eur. J. Pediatr.*, **155**, 262.
5. Poggi, F., Rabier, D., Vassault, A., Charpentier, C., Kamoun, P. and Saudubray, J. M., 1994, Protocole d'investigations métaboliques dans les maladies héréditaries du metabolisme. *Arch. Pediatr.*, **1**, 667.
6. Galán, A., Coll, J., Padròs, A., Arambarri, M. and Pintos, G., 1999, Estrategia diagnòstica de las enfermedades mitocondriales. *Rev. Neurol.*, **29**, 52.
7. Bonnefont, J. P., Specola, N. B., Vassault, A., Lombes, A., Ogier, H., de Klerk, J. B. C. *et al.*, 1990, The Fasting test in paediatrics:

application to the diagnosis of pathological hypo and hyperketonic states. *Eur. J. Pediatr.*, **150**, 80.

8. Williamson, D. H. and Mellanby, J., 1974, D-3-Hydroxybutyrate. In: H. U. Bergmeyer (ed.) *Methods of Enzymatic Analysis* (New York: Academic Press), p. 1836.
9. Mellanby, J. and Williamson, D. H., 1974, Acetoacetate. In: H. U. Bergmeyer (ed.) *Methods of Enzymatic Analysis* (New York: Academic Press), p. 1840
10. Nuwayhid, N. F., Johnson, G. F. and Feld, R. D., 1989, Multipoint kinetic method for simultaneously measuring the combined concentrations of acetoacetate-β-hydroxybutyrate and lactate-pyruvate. *Clin. Chem.*, **35**, 1526.
11. Hansen, J. L. and Freier, E. F., 1978, Direct assays of lactate, pyruvate, β-hydroxybutyrate and acetoacetate with a centrifugal analyzer. *Clin. Chem.*, **24**, 475.
12. Li, P. K., Lee, J. T., MacGillivray, M. H., Schaefer, P. A. and Siegel, J. H., 1980, Direct, fixed-time kinetic assays for β-hydroxybutyrate and acetoacetate with a centrifugal analyzer or a computer-backed spectrophotometer. *Clin. Chem.*, **26**, 1713.
13. Artuch, R., Vilaseca, M. A, Farré, C. and Ramon, F., 1995, Determination of lactate, pyruvate, β-hydroxybutyrate and acetoacetate with a centrifugal analyser. *Eur. J. Clin. Chem. Clin. Biochem.*, **33**, 529.
14. Uno, S., Takehiro, O., Tabata, R. and Ozawa, K., 1995, Enzymatic method for determining ketone body ratio arterial blood. *Clin. Chem.*, **41**, 1745.
15. Mitchell, G. A., Kassovska-Bratinova, S., Boukaftane, Y., Robert, M.-F., Wang, S. P., Ashmarina, L., Lambert, M., Lapierre, P. and Potier, E., 1995, Medical aspects of ketone body metabolism. *Clin. Invest. Med.*, **18**, 193.
16. Vassault, A., Bonnefont, J. P., Specola, N. and Saudubray, J. M., 1991, *Lactate, Pyruvate and Ketone Bodies. Techniques in Diagnostic Human Biochemical Genetics: A Laboratory Manual* (New York: Wiley-Liss) p. 285.
17. Societé Française de Biologie Clinique, 1986, Commission 'Validation de techniques'. Protocole de validation de techniques. *Ann. Biol. Clin.*, **44**, 686.
18. European Committee for Clinical Laboratory Standards, 1986, Guidelines for the evaluation of analyzers in clinical chemistry. ECCLS Document, **3**(2).
19. International Federation of Clinical Chemistry, 1987, Approved recommendation (1987) on the theory of reference values. Part 5. Statistical treatment of collected reference values. Determinations of reference limits. *Clin. Chem. Acta*, **S13-S32**, 170.
20. Lamers, K. J. B., Doesburg, W. H., Gabreëls, F. J. M., Romsom, A. C., Renier, W. O., Wevers, R. A. and Lemmens, W. A., 1985, Reference values of blood components related to fuel metabolism in children after an overnight fast. *Clin. Chim. Acta*, **145**, 17.

